# No evidence of durable trained immunity after two doses of adenovirus-vectored or mRNA COVID-19 vaccines

**DOI:** 10.1172/JCI171742

**Published:** 2023-09-01

**Authors:** Natalie E. Stevens, Feargal J. Ryan, Nicole L. Messina, Stephen J. Blake, Todd S. Norton, Susie Germano, Jane James, Georgina L. Eden, Yee C. Tee, Miriam A. Lynn, Rochelle Botten, Simone E. Barry, Nigel Curtis, David J. Lynn

**Affiliations:** 1 Precision Medicine Theme, South Australian Health and Medical Research Institute, Adelaide, South Australia, Australia.; 2 Flinders Health and Medical Research Institute, Flinders University, Bedford Park, South Australia, Australia.; 3 Murdoch Children’s Research Institute, Royal Children’s Hospital, Parkville, Victoria, Australia.; 4 Department of Paediatrics, University of Melbourne, Parkville, Victoria, Australia.; 5 Department of Thoracic Medicine, Royal Adelaide Hospital, Adelaide, South Australia, Australia.

**Keywords:** COVID-19, Vaccines, Epigenetics, Innate immunity, Monocytes

**To the Editor:** Trained immunity (TI) is defined as the long-term metabolic and epigenomic reprogramming of innate immune cells, priming them for enhanced responses to subsequent challenges, including unrelated infections (1). Recently, Murphy et al. (2) reported in the *JCI* that three months after one dose of the ChAdOx1-S (Oxford/AstraZeneca) adenovirus-vectored SARS-CoV-2 vaccine, multiple changes consistent with TI were observed in a cohort of 10 individuals. These changes included an increased frequency of circulating monocytes, enhanced monocyte activation marker expression, and increased cytokine and chemokine responses. Whether these changes were accompanied by epigenomic reprogramming of monocytes, a hallmark of TI, was not assessed. In contrast, Yamaguchi et al. (3) reported only transient epigenomic and transcriptomic changes in monocytes collected from five individuals following two doses of the BNT162b2 (Pfizer/BioNTech) mRNA vaccine. These findings suggest that ChAdOx1-S but not BNT162b2 vaccination induces TI, which could have important implications for the use of COVID-19 vaccines globally (4).

In our study, we assessed whether two doses of the BNT162b2 or ChAdOx1-S vaccines induced altered innate immune responses or epigenomic changes consistent with TI in a cohort of 46 healthy adults (Supplemental Table 1; supplemental material available online with this article; https://doi.org/10.1172/JCI171742DS1) recruited as part of the COVID-19 Vaccine Immune Responses Study (5). Baseline characteristics of ChAdOx1-S (*n* = 13) and BNT162b2 (*n* = 33) recipients were not significantly different. PBMCs were collected before vaccination and 28.1 ± 1.7 days following the second dose of either ChAdOx1-S or BNT162b2 (Figure 1A and Supplemental Methods). As heightened inflammatory cytokine responses are a hallmark of TI, we first assessed cytokine responses in PBMCs following stimulation with a range of bacterial, viral, and fungal stimulants. Cytokine responses were not significantly increased following two doses of either vaccine compared with prevaccination responses (Figure 1B and Supplemental Figure 1). Furthermore, surface expression of activation markers (CD86 and HLA-DR) on CD14^+^CD16^–^ monocytes (Figure 1C) as well as total CD14^+^ monocytes (Supplemental Figure 2A) was not significantly altered compared with prevaccination samples. These data contrast with the recent report of significantly increased monocyte cytokine responses and activation marker expression after one dose of the ChAdOx1-S vaccine (2). We also assessed PBMC cytokine responses following stimulation with inactivated SARS-CoV-2 viral supernatant, which stimulates both innate and antigen-specific cytokine responses. Significantly higher IFN-γ release (*P* = 0.0092) was observed after two doses of BNT162b2 but not ChAdOx1-S (Supplemental Figure 1). These findings align with our previous assessment of adaptive immune responses in this cohort, which showed that antibody and CD4^+^ T cell responses were significantly lower in ChAdOx1-S compared with BNT162b2 recipients (5).

Altered chromatin accessibility has been shown to accompany the epigenomic reprogramming associated with vaccine-induced TI (6). To assess this, we sorted classical monocytes (CD14^+^CD16^–^) from participants before and after vaccination and performed ATAC-Seq (*n* = 82, mean 63M reads/sample; Supplemental Table 2). The ATAC-Seq data were of high quality, with sequencing depth and enrichment of reads near transcription start sites (TSSs) conforming to ENCODE data standards (Supplemental Figure 2, C–E, and Supplemental Table 2). Dimensionality reduction analysis revealed, however, that pre- and postvaccination samples did not cluster separately (Figure 1D). Consistent with these data, we did not detect any differentially accessible regions (Figure 1, E and F, Supplemental Figure 2F, and Supplemental Table 2) or significantly enriched pathways after vaccination, even when using a less stringent statistical threshold (FDR of 10%). Together, these data indicate that chromatin accessibility in monocytes is not significantly altered 28 days after two doses of either vaccine.

Our data suggest that long-term TI is not induced in human PBMCs or circulating classical monocytes following two doses of either the ChAdOx1-S or BNT162b2 vaccines. Further investigation is needed to assess whether these vaccines have any effects on TI induced in cell types not analyzed here (e.g., granulocytes). Our data, which are from a larger cohort, are consistent with the conclusion of Yamaguchi et al. (3) that BNT162b2 vaccination does not induce long-term epigenetic reprogramming of monocytes. Why our data contrast with the evidence of TI after one dose of ChAdOx1-S reported by Murphy et al. (2) is currently unclear. Methodological differences or our modestly larger sample size of ChAdOx1-S recipients could potentially explain these discrepancies; however, we believe that the most likely difference is that we investigated samples collected 28 days after two doses of ChAdOx1-S while Murphy et al. investigated samples collected after only one dose. Our data suggest that any effects of these vaccines on TI after one dose are transient and not induced after a second dose, which is important given that the vast majority of people have received these vaccines as multidose regimens. Our findings therefore have important implications for current and future mRNA and adenoviral-vectored vaccines and support the safety of these vaccine technologies.

## Supplementary Material

Supplemental data

Supplemental table 2

Supporting data values

## Figures and Tables

**Figure 1:**
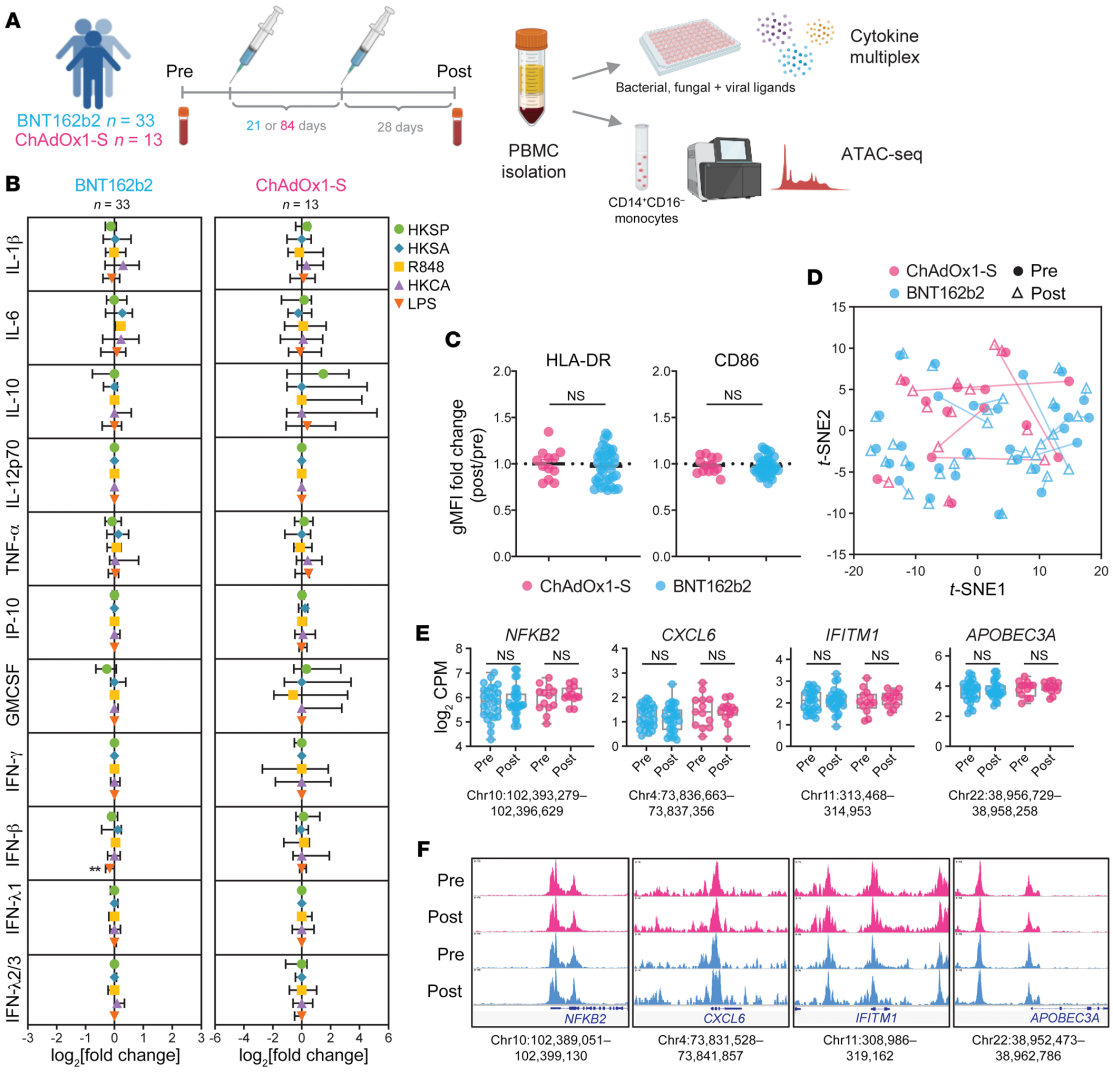
No evidence of trained immunity in PBMCs or circulating monocytes after two doses of ChAdOx1-S or BNT162b2 vaccines. (**A**) PBMCs collected from participants before vaccination (Pre) and approximately 28 days following (Post) the second dose of the BNT162b2 (*n* = 33) or ChAdOx1-S (*n* = 13) vaccines were stimulated in vitro with heat-killed *Streptococcus pneumoniae* (HKSP), heat-killed *Staphylococcus aureus* (HKSA), resiquimod (R848), heat-killed *Candida albicans* (HKCA), or LPS for 20 to 22 hours. Cytokine production was quantified via multiplex immunoassay. (**B**) Shown is log_2_ fold-change (median ± 95% CI) in cytokine concentrations (after versus before vaccination). (**C**) Expression (geometric mean fluorescence intensity [gMFI]) of HLA-DR and CD86 on classical monocytes (before versus after vaccination). (**D**) Shown is t-distributed stochastic neighbor embedding (*t*-SNE) analysis of ATAC-Seq data before and after vaccination. (**E**) Mean chromatin accessibility and (**F**) representative plots of peaks ± 5 kb of the TSS of selected genes previously shown to have altered accessibility in monocytes following BNT162b2 or BCG vaccination (4, 6). CPM, counts per million. (**B**, **C,** and **E**) Wilcoxon’s log-rank tests (after versus before vaccination). (**C**) Groups compared via Mann-Whitney *U* test. **P < 0.01.

## Version 1 07/20/2023

In-Press Preview

## Version 2 09/01/2023

Electronic publication

## Version 3 09/05/2023

Corrected labels in figure 1
