# Supplementary material for: No evidence of durable trained immunity after two doses of adenovirus-vectored or mRNA COVID-19 vaccines
Source: J Clin Invest. 2023 Sep 1;133(17):e171742. doi: 10.1172/JCI171742 (PMC10471164; doi:10.1172/JCI171742)
Supplement: Supplemental data [file jci-133-171742-s014.pdf]

Supplementary Material

for

"No evidence of durable trained immunity after two doses of adenovirus-vectored or mRNA COVID-19 vaccines"

Contents

Supplementary Materials and Methods 1
- Subject details 1
- Blood collection and cryopreservation 1
- PBMC thawing 2
- PBMC stimulation 2
- Cytokine quantitation 3
- Monocyte sorting 3
- ATAC-seq transposition 4
- ATAC-seq library preparation and sequencing 5
- ATAC-seq data analysis 6
- Statistics 7
- Study Approval 8
- Data availability 8

Supplementary Table S1: Participant demographic data 9
Supplementary Figure S1: Cytokine concentrations following in vitro stimulation of PBMC collected pre- and post-vaccination. 10
Supplementary Figure S2: Supplementary flow cytometry and ATAC-seq analysis 12
Acknowledgements 14
Authorship Contributions 14
Supplementary References 15

Supplementary Materials and Methods

Subject details

Participants included in this study were a subset of the participants recruited to the COVID-19 Vaccine Immune Responses Study (COVIRS), which has been described previously (1). One participant was recruited under the 'BCG vaccination to reduce the impact of COVID-19 in healthcare workers (BRACE)' trial protocol. Samples from this individual were processed in the same way as other COVIRS participants (2). Participants were healthy, SARS-CoV-2 naïve adults (63% female, mean age 41 years) who received either two doses of the BNT162b2 vaccine approximately 3 weeks apart (n=34) or two doses of the ChAdOx1-S (n=13) vaccine approximately 3 months apart (Table S1). Age, sex, body mass index, smoking and alcohol use status, and reported underlying health issues were not significantly different between ChAdOx1-S and BNT162b2 recipients. Samples were collected in South Australia in 2021 where strict border controls prevented community transmission of SARS-CoV-2 in the State. Participants provided a blood sample 1-14 days prior to their 1st COVID-19 vaccine (Pre; mean 6.3 days prior to dose 1) and ~28 days after the 2nd dose (Post, mean 28.1 days after dose 2). Blood samples were processed within 3 hours of collection, by a dedicated team using a standardised protocol.

Blood collection and cryopreservation

Blood collected into sodium heparin tubes was used for isolation of peripheral blood mononuclear cells (PBMC), as described previously (1). Briefly, sodium heparin anticoagulated blood was diluted 1:1 with PBS and subjected to density gradient centrifugation (Lymphoprep, Stem Cell Technologies). PBMC were isolated from the

interphase via manual pipetting, washed twice in PBS and resuspended in freezing medium (10% DMSO, Sigma-Aldrich & 90% FCS, CellSera). Aliquots of cells (500μL) were frozen using a Mr. Frosty™ Freezing Container (Nalgene) in a -80°C freezer prior to transfer to liquid nitrogen for longer-term storage.

PBMC thawing

Samples collected pre- and post-vaccination were thawed, processed and analysed on the same day in batches of 5-10 participants. On the day of analysis, frozen cryovials of cells were rapidly thawed in a 37°C water-bath. Pre-warmed RPMI medium (Sigma-Aldrich) with 10% FCS (CellSera) was added (1mL) dropwise to thawed cells, and cells were pipetted into 5mL of prewarmed RPMI with 10% FCS. Cells were centrifuged (180 x *g*, 10 minutes, at RT) and washed twice prior to downstream analysis.

PBMC stimulation

Following thawing, 180μL of PBMC (~200,000 cells/well) were plated into pre-aliquoted round-bottom strip wells containing 20μL media or one of the following immune stimulants: heat killed *C. albicans* (HKCA; *C. albicans* 11145487; 10^7 cells/well), resiquimod (R848; 35μg/mL; Invivogen), lipopolysaccharide (LPS; 100ng/mL; *E. coli* serotype 055:B5, Sigma-Aldrich), heat-killed *Staphylococcus aureus* (HKSA; *S. aureus* 11191224, 10^8 cells/well), heat-killed *Streptococcus pneumoniae* (HKSP; *S pneumoniae* HK 12095233, 10^8 cells/well), γ-irradiated SARS-CoV-2 culture supernatant (iSARS; 104.7 $TCID_{50}$/ml, prepared from VIC01 virus, NCBI: MT007544.1) or Vero cell control supernatant. HKCA, HKSA and HKSP were clinical

isolates from children with invasive disease at the Royal Children's Hospital Melbourne and were killed by heat treatment at 70°C for 2 hours, as previously described (3). iSARS supernatant was kindly provided by Prof. Kanta Subbarao and Dr Rajeev Rudraraju and prepared as previously described (4) from virus provided by the Victorian Infectious Diseases Reference Laboratory. PBMC were cultured for 20-22 hours at 37°C with and without stimulation and supernatants stored at -80°C prior to analysis.

Cytokine quantitation

A flow cytometric bead multiplex assay (LEGENDplex™ Human Anti-Virus Response 13-plex Panel, Biolegend) was used to quantify 13 cytokines (IL-1β, IL-6, IL-8, IL-10, IL-12p70, IFN-α, IFN-β, IFN-λ1, IFN-λ2/3, IFN-γ, TNF-α, IP-10/CXCL10 & GM-CSF) in supernatants collected as described above according to the manufacturer's instructions, with modifications as previously described (1). Samples were diluted 1:10 prior to analysis. PE fluorescence (geometric mean fluorescence intensity) was determined for each sample and cytokine concentrations were interpolated from a standard curve (sigmoidal 4-parameter logistic curve) using GraphPad Prism 9 software. Undetectable (low) readings were adjusted to the minimum detection threshold of the assay. As the majority of readings for IL8 and IFN-α were above and below the standard curve, respectively, these data were not analysed further.

Monocyte sorting

Thawed PBMC were stained and used for monocyte isolation via Fluorescence-Activated Cell Sorting (FACS). Cells were pelleted and resuspended in 25μL Brilliant

Stain Buffer (BD) containing 1:20 Fc Block (Human Trustain FCX, Biolegend, 422301) and 1:500 viability dye (eflour 506, Thermo Fisher, 65-0866-14) for 10 minutes prior to the addition of 25μL surface staining cocktail containing CD19 PE (BD 555413; 1:10), CD3 FITC (BD 561802; 1:25), CD56 Pe-Cy7 (BD 362509; 1:25), HLA-DR APC-H7 (BD 561358; 1:50), CD86 PerCPCy5.5 (BD 561129; 1:50), CD16 BV421 (BD 562878; 1:50), CD14 A647 (BD 562690; 1:50) in FACS buffer (0.1% BSA, 2mM EDTA in PBS). Following 30 minutes incubation on ice, cells were washed with 5mL FACS buffer, centrifuged (300 x *g* for 5 minutes at 4°C) and resuspended in 500 μL FACS buffer for sorting. Data were acquired on a BD FACSMelody or BD FACSAria Fusion, and classical monocytes (defined as live $CD19^{-}CD3^{-}CD56^{-}CD14^{+}CD16^{-}$ cells) were sorted into RPMI containing 10% FCS (CellSera) for ATAC-seq. Data were analysed using FlowJo v10.8. Geometric mean fluorescence intensity of APC-H7 and PerCPCy5.5 were exported to assess HLA-DR and CD86 expression, respectively.

ATAC-seq transposition

Monocytes were transposed for ATAC-seq using the omni-ATAC method (5, 6). Briefly, 50,000 cells were centrifuged (500 x *g*) and thoroughly resuspended in 50μL cold lysis buffer (ATAC Resuspension Buffer containing 0.1% Tween-20, 0.1% NP40, and 0.01% Digitonin) before 3 minutes incubation on an ice block. Samples were washed out with 450μL ATAC Resuspension buffer with 0.1% Tween-20, and nuclei pelleted via centrifugation (600 x *g* for 10 min at 4°C) prior to addition of 40μL freshly prepared transposition mixture (containing 2μL Tn5 transposase, 0.01% Digitonin and 0.1% Tween-20). Samples were incubated at 37°C for 40 minutes in a Thermomixer (Eppendorf; 1000 RPM). Following transposition, 250μL DNA binding buffer from the Clean and Concentrator-5 kit (Zymo, D4014) was added and reactions stored at -80°C.

ATAC-seq library preparation and sequencing

Transposed DNA was cleaned using the Clean and Concentrator-5 kit (Zymo, D4014) according to the manufacturer's instructions, and eluted in 21µL of elution buffer. DNA was subjected to PCR amplification (72°C 5 min, 98°C 30 sec, then 8-15 cycles of 98°C 10 sec, 63°C 30 sec and 72°C 1 min) with adaptor-specific primers containing Illumina index barcode sequences, as previously described (5, 6). Amplified libraries were cleaned up using Zymo DNA Clean and Concentrator 5 columns followed by RHS clean-up with SPRIselect beads (Beckman Coulter) to deplete large molecular weight fragments >1000bp. For pre-sequencing quality control (QC), PCR was performed on libraries to assess enrichment of accessible regions, selected manually from publicly available ATAC-seq data (7).

Accessible regions were:

115bp sequence within an enhancer region of *ACTB*

F: 5' AAAAGGCAAACACTGGTCGG 3'

R: 5' GACCCGGCGCTGTTTGAA 3'

140bp sequence within the promotor region of *VCP*

F: 5' ACACGCCTAGTAACGCCAC 3'

R: 5' GAGTCCGCCTCTAAGGGAT 3'

Inaccessible regions were:

104bp sequence within an intragenic region of *REEP5*

F: 5’ GGTCTGTGAGGGAGAAATGGG 3’

R: 5’ GGGAGTTGTGAGGTTGCCTG 3’

107bp sequence within exon 4 of *VSP29*

F: 5’ CACTGTTAATGTCGGCATGGTAT 3’

R: 5’ AAAGTGGACGAGATGACCTTTCA 3’

qPCR was performed using 5μL of 1:10 diluted library using the PowerUp SYBR Green Master Mix (Applied Biosystems) according to the manufacturer’s instructions. Genomic DNA (gDNA; 1ng/μL) was run as a comparison. The accessibility ratio was calculated as the mean copy number of accessible regions divided by the mean copy number of inaccessible regions, relative to gDNA. Enrichment was >1 for all samples. Libraries were visualised using a BioAnalyzer instrument (Agilent) using the High Sensitivity DNA Assay (Agilent) kit, according to the manufacturer’s instructions, to confirm that fragment sizes were consistent with high-quality ATAC-seq libraries (**Fig. S2C**), and to determine library concentration. Following QC, libraries were sequenced on a NovaSeq 6000 sequencer (Illumina) S4 Flow Cell (2 x 150bp PE reads).

ATAC-seq data analysis

Fastq read quality was assessed using FastQC version v0.12.1 and summarized with MultiQC (8). Adapter sequences were removed using Cutadapt v4.2 (9), and remaining sequences were quality filtered with Trimmomatic v0.39 (10) (sliding (4nt) window with a minimum PHRED score of 20, together with average score of 30).

Reads were then aligned to the GRCh38 human genome using Bowtie v2.5.1 "–very-sensitive" mode (11). SAMtools v1.6 was used to convert to BAM format and sort alignments (12). The Bioconductor package ATACseqQC (v1.22.0) was used to assess sample quality and library complexity (7). Peaks were called across all samples using Genrich v0.6.1 in the 'ATAC-seq mode' with a q value threshold of 0.05, excluding mitochondrial peaks and removing PCR duplicates. Feature Counts v2.0.4 was used to count aligned reads to generate a count per sample for each peak (13). These counts were then imported into R v4.2.0 for further statistical analysis. Peaks were annotated with ChIPseeker v1.32.1. Peaks were filtered to only include those with at least 100 reads uniquely aligned in at least 8 samples. EdgeR (v3.40.2) was used to normalize the data (using trimmed mean of M-values method) and perform differential accessibility analyses (with the glmLRT function) (14). QC metrics (Table S2) were assessed as described in ENCODE consortium guidelines (Available at https://www.encodeproject.org/atac-seq/), including sequencing depth (100% of samples had >25 million paired-end reads), alignment rate (100% of samples had alignment rates of >98%) transcription start site enrichment (100% of samples 'acceptable' or 'ideal'), non-redundant fraction (NRF; >98% samples 'acceptable' or 'ideal'), PCR bottlenecking coefficients (100% of samples 'acceptable' or 'ideal') and fraction of reads in called peaks (FRiP score; 100% of samples >0.3). Peaks ±5kb of the TSS of selected genes were visualized using the Integrative Genomics Viewer v2.10.0 06.

Statistics

Cytokine concentrations and activation marker staining between pre- and post-vaccination samples were assessed via a Wilcoxon matched-pair signed rank test

using GraphPad Prism V9.0 software. A P value less than 0.05 was considered statistically significant. Potential differentially accessible regions (DARs) were assessed between pre- and post-vaccination samples for each vaccine separately (model ~ Participant + Timepoint) and by comparing between vaccines over time in a nested comparison (model ~ Participant + Timepoint:Vaccine). Neither approach identified any statistically significant DARs with a FDR < 0.1. Enrichment of REACTOME and Gene Ontology (GO) pathways/terms was assessed among the top 500 regions ranked by unadjusted P value using a hypergeometric test. Regions were split according to positive log2 fold changes (i.e., peaks with increased accessibility) or negative log2 fold changes (i.e. decreased accessibility). In order to ensure enrichment analyses were robust (as none of these regions individually achieved FDR < 0.1), 250 regions were randomly picked and tested for enrichment of pathways/GO terms 1000 times. Any pathways/GO terms found enriched in the randomly selected subsets were not considered to be significantly enriched.

Study Approval

Participants were recruited in Adelaide, South Australia under protocols approved by the CALHN Human Research Ethics Committee, Adelaide, Australia (Approval No. 14778) or by the Royal Children's Hospital, Melbourne, Australia, Human Research Ethics Committee (Approval No. 62586), in accordance with national and international guidelines. All participants provided written informed consent prior to commencement of study procedures.

Data availability

Cytokine concentrations from stimulation assays and mean fluorescence (geometric mean) of monocyte activation markers are provided as Supporting Data Values. The ATAC-seq sequencing data reported in this paper is available at the Gene Expression Omnibus under accession number GSE225165.

Table S1: Participant demographic data

	**ChAdOx1-S**	**BNT162b2**
Number of participants	13	33
Age (years)	mean=45.8, SD=15.6	mean=38, SD=11.1
Sex	54% female	62% female
Body Mass Index	Mean=27, SD=6	Mean=26, SD=5.12
Smoker	76% non-smoker	91% non-smoker
Alcohol intake	16% non-drinkers	39% non-drinkers
Any underlying health issues? (e.g. hypertension, asthma)	15% report health issue	12% report health issue

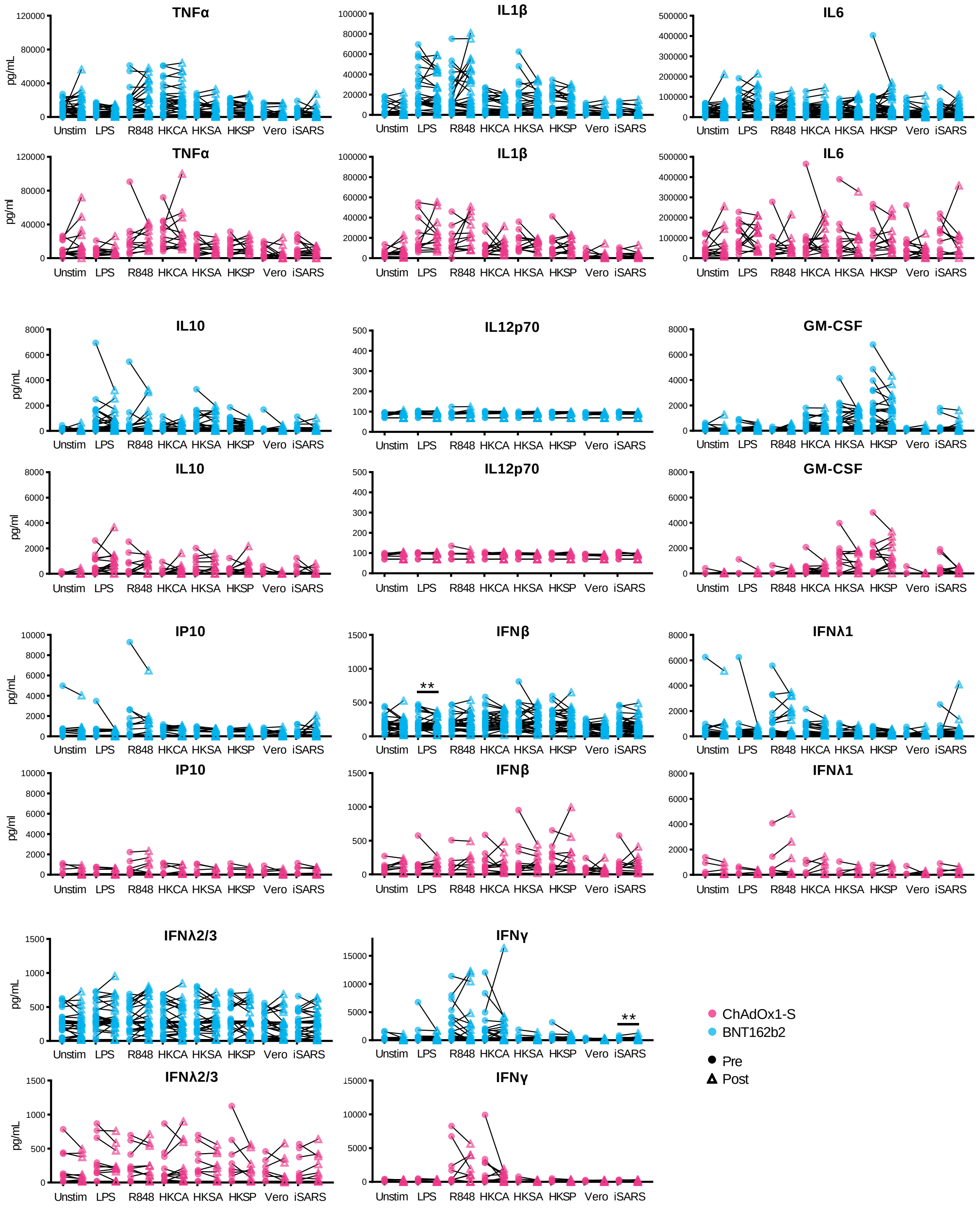

Figure S1: Cytokine concentrations following *in vitro* stimulation of PBMC collected pre- and post-vaccination. PBMC were collected from participants pre-vaccination and ~28 days following the second dose of the BNT162b2 (n=34) or ChAdOx1-S (n=12) vaccines and were either unstimulated or stimulated *in vitro* with lipopolysaccharide (LPS), resiquimod (R848), heat-killed *C. albicans* (HKCA),

heat-killed *S. aureus* (HKSA), heat-killed *S. pneumoniae* (HKSP), Vero cell supernatant (control for iSARS stimulation), or γ-inactivated SARS-CoV-2 supernatant (iSARS), for 20-22 hours. Cytokines were quantified via multiplex bead array. The concentration of TNFα, IL1β, IL6, IL10, IL12p70, GM-CSF, IP10, IFNβ, IFNλ1, IFNλ2/3 & IFNγ in supernatants from BNT162b2 (blue) and ChAdOx1-S (pink) recipients are shown. Statistical significance was assessed using Wilcoxon signed-rank tests. ** $P<0.01$.

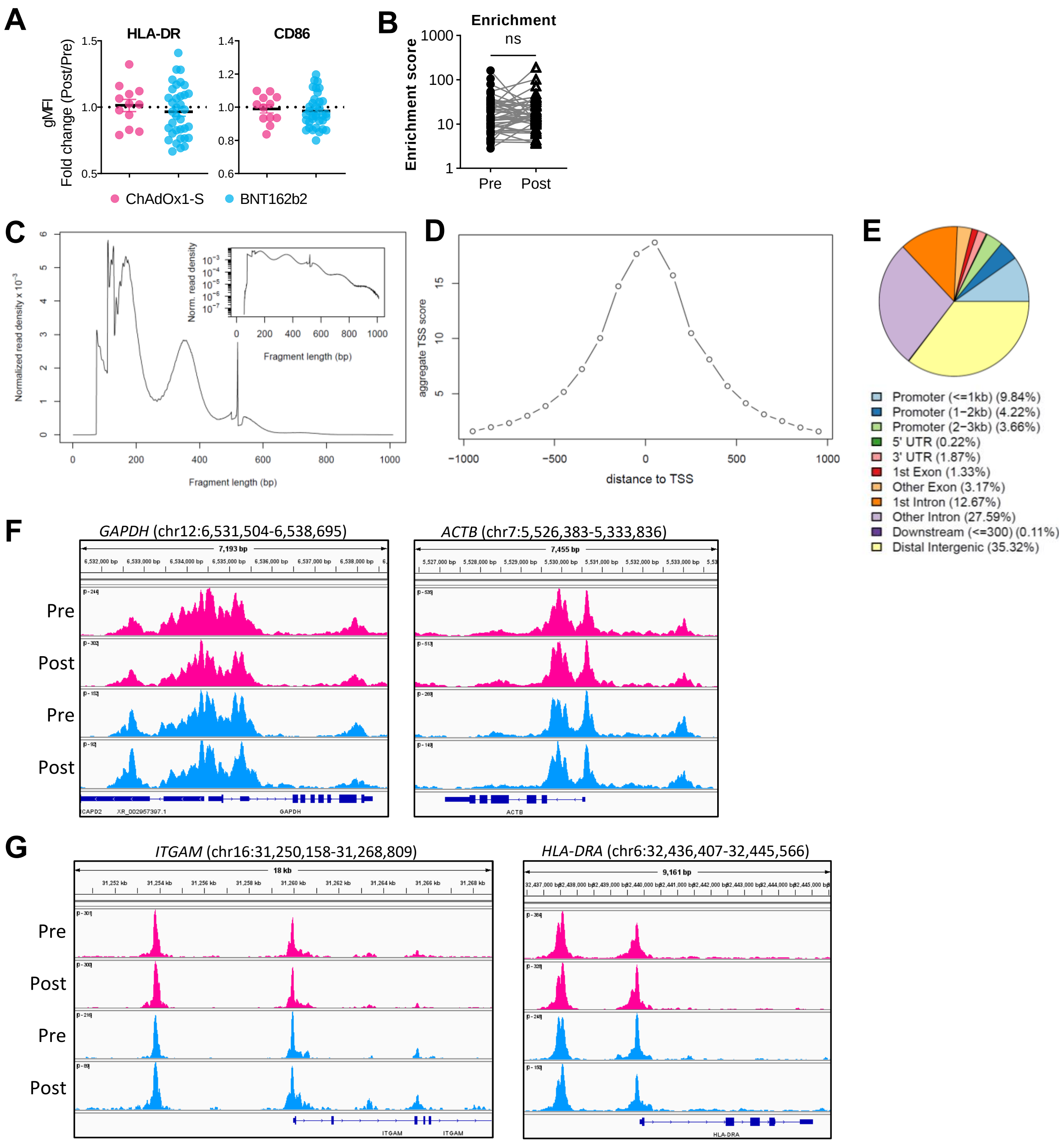

Figure S2: Supplementary flow cytometry and ATAC-seq analysis. PBMC from 46 participants (33 BNT162b2 recipients; 13 ChAdOx1-S recipients) pre- and post-vaccination were assessed via flow cytometry. (**A**) Geometric mean fluorescence intensity (gMFI) of HLA-DR and CD86 on total CD14$^+$ monocytes relative to pre-vaccination levels. ATAC-seq was performed on sorted classical monocytes (CD14$^+$CD16$^-$) isolated from 41 participants (28 BNT162b2 recipients; 13 ChAdOx1-S recipients) pre-

and post-vaccination. **(B)** Enrichment of accessible compared to inaccessible regions in pre- and post-vaccination samples, as determined by qPCR. **(C)** Size distribution of sequenced fragment size for one representative sample. **(D)** Transcription start site (TSS) enrichment for one representative sample as calculated by ATACSeqQC. See Table S2 for QC data for all samples. **(E)** Peak annotation generated by ChIPseeker. **(F)** Chromatin accessibility at housekeeping genes *GAPDH* and *ACTB* in a ChAdOx1-S-vaccinated participant (pink) and a BNT162b2 vaccinated participant (blue) pre- and post-vaccination. **(G)** Chromatin accessibility at *ITGAM* (encoding CD14) and *HLA-DRA* in a ChAdOx1-S-vaccinated participant (pink) and a BNT162b2 vaccinated participant (blue) pre- and post-vaccination. These loci have previously been reported to be accessible in human monocyte/myeloid cell populations (15). (**B**) Statistical significance assessed using Wilcoxon signed-rank testing.

Acknowledgements

The authors thank all of the study participants. This study was supported by funding from Bioplatforms Australia, the Flinders Foundation, NHMRC and EMBL Australia Group Leader funding. Flow cytometry analysis and cell sorting was performed at the ACRF Cellular Imaging and Cytometry Core Facility in SAHMRI. We thank the South Australian Genomics Centre (SAGC) for the sequencing of ATAC-seq libraries. The SAGC is supported by the National Collaborative Research Infrastructure Strategy (NCRIS) via BioPlatforms Australia and by the SAGC partner institutes. The authors thank Prof. Kanta Subbarao and Dr Rajeev Rudraraju (both affiliated with the Peter Doherty Institute for Infection and Immunity and University of Melbourne, Melbourne, Australia) for providing iSARS supernatant for use in this study.

Author Contributions

DJL conceived of and supervised the study. NES designed and performed the experiments and analysed cytokine data. FJR performed ATAC-seq data analysis. NLM, SG and NC provided the microbial ligands for PBMC stimulations and consulted on study design. SJB and TSN assisted with cell thawing and sorting. JJ, GLE, YCT and MAL co-ordinated sample receipt, processing and storage. RB co-ordinated sample collection with participants. SEB and NC contributed to study design of COVIRS. The manuscript was written by NES, FJR and DJL with approval from all authors.

Supplementary References

1. Ryan FJ, Norton TS, McCafferty C, Blake SJ, Stevens NE, James J, et al. A systems immunology study comparing innate and adaptive immune responses in adults to COVID-19 mRNA and adenovirus vectored vaccines. Cell Reports Medicine. 2023.
2. Pittet LF, Messina NL, Gardiner K, Orsini F, Abruzzo V, Bannister S, et al. BCG vaccination to reduce the impact of COVID-19 in healthcare workers: Protocol for a randomised controlled trial (BRACE trial). BMJ Open. 2021;11(10):e052101.
3. Freyne B, Donath S, Germano S, Gardiner K, Casalaz D, Robins-Browne RM, et al. Neonatal BCG Vaccination Influences Cytokine Responses to Toll-like Receptor Ligands and Heterologous Antigens. J Infect Dis. 2018;217(11):1798-808.
4. Messina NL, Germano S, McElroy R, Rudraraju R, Bonnici R, Pittet LF, et al. Off-target effects of bacillus Calmette-Guerin vaccination on immune responses to SARS-CoV-2: implications for protection against severe COVID-19. Clin Transl Immunology. 2022;11(4):e1387.
5. Corces MR, Trevino AE, Hamilton EG, Greenside PG, Sinnott-Armstrong NA, Vesuna S, et al. An improved ATAC-seq protocol reduces background and enables interrogation of frozen tissues. Nat Methods. 2017;14(10):959-62.
6. Stevens NE, van Wolfswinkel M, Bao W, Ryan FJ, Brook B, Amenyogbe N, et al. Immunisation with the BCG and DTPw vaccines induces different programs of trained immunity in mice. Vaccine. 2021.
7. Ou J, Liu H, Yu J, Kelliher MA, Castilla LH, Lawson ND, et al. ATACseqQC: a Bioconductor package for post-alignment quality assessment of ATAC-seq data. BMC Genomics. 2018;19(1):169.
8. Ewels P, Magnusson M, Lundin S, Käller M. MultiQC: summarize analysis results for multiple tools and samples in a single report. Bioinformatics. 2016;32(19):3047-8.
9. Martin M. Cutadapt removes adapter sequences from high-throughput sequencing reads. EMBnet journal. 2011;17(1):10-2.

10. Bolger AM, Lohse M, Usadel B. Trimmomatic: a flexible trimmer for Illumina sequence data. Bioinformatics. 2014;30(15):2114-20.

11. Langmead B, Salzberg SL. Fast gapped-read alignment with Bowtie 2. Nature methods. 2012;9(4):357-9.

12. Li H, Handsaker B, Wysoker A, Fennell T, Ruan J, Homer N, et al. The sequence alignment/map format and SAMtools. Bioinformatics. 2009;25(16):2078-9.

13. Liao Y, Smyth GK, Shi W. featureCounts: an efficient general purpose program for assigning sequence reads to genomic features. Bioinformatics. 2014;30(7):923-30.

14. Robinson MD, McCarthy DJ, Smyth GK. edgeR: a Bioconductor package for differential expression analysis of digital gene expression data. Bioinformatics. 2010;26(1):139-40.

15. Satpathy AT, Granja JM, Yost KE, Qi Y, Meschi F, McDermott GP, et al. Massively parallel single-cell chromatin landscapes of human immune cell development and intratumoral T cell exhaustion. Nat Biotechnol. 2019;37(8):925-36.
